# Diseases of the Nose, Throat and Ear

**Published:** 1908-10

**Authors:** 


**Diseases of the Nose, Throat and Ear.** Medical and Surgical. By **William Lincoln Ballenger,** M.D., Professor of Otology, Rhinology and Laryngology, College of Physicians and Surgeons, of Chicago University of Illinois. Octavo, 896 pages, with 467 engravings and 16 plates. Lea & Febiger, Publishers, Philadelphia and New York, 1908. (Cloth, $5.50 net.)

It is a genuine pleasure to examine such a book as this, one so filled with scientific material, one which constitutes a valuable contribution to the literature of medicine. Its first notable feature is that it includes in its delineation the nose, throat, and ear in their entirety. Heretofore it has been customary for writers to deal fully, even amply with the nose and throat, but at most with the associated affections of the ear. Ballenger has introduced also the relationship of the eye to the diseases of the sinuses, though appreciative of the fact that the significance of this relationship is little understood. A marked feature of this work is the illustrations, many of which are original while all are well executed, making an instructive and clear exposition of the author's meaning and of his work. Especially is Dr. Ballenger interesting in dealing with diseases of the maxillary and accessory sinuses. Every laryngologist should possess the book.

